# Multi-Source Visual Fusion for Face-Guard Pose Estimation and Shearer Cutting-Interference Warning in Fully Mechanized Coal Mining Faces

**DOI:** 10.3390/s26196082

**Published:** 2026-09-25

**Authors:** Yihui Zhao, Zhongbin Wang, Dan Yang, Dong Wei

**Affiliations:** 1 School of Mechatronic Engineering, China University of Mining and Technology, No. 1 Daxue Road, Xuzhou 221116, China; lb22050023@cumt.edu.cn (Y.Z.); wangzbpaper@126.com (Z.W.); 6297@cumt.edu.com (D.Y.); 2 Xi’an Coal Mining Machinery Co., Ltd., No. 7 Jingpu Road, Gaoling District, Xi’an 710032, China

**Keywords:** multi-source visual fusion, face-guard pose estimation, shearer cutting interference, infrared imaging, LiDAR, coal mining safety

## Abstract

Fully mechanized coal faces require robust sensing of hydraulic-support face-guard pose and its clearance to the shearer drum under dust, mist, and low-light conditions. This paper presents a multi-source visual fusion framework that integrates an RGB camera, an infrared camera, and a 32-line LiDAR. The system performs modality-specific denoising, distortion correction, time synchronization, spatial registration, and adaptive feature fusion before estimating face-guard 2D boxes, 3D keypoints, and 6DoF pose. RGB and infrared features are extracted with Swin Transformer backbones, LiDAR features are encoded in BEV space, and the fused representation is refined by an axial cross-modal fusion block. The recovered pose is converted into a 3D envelope, and the minimum distance to the shearer drum is mapped to a four-level warning policy. Experiments on 2400 synchronized multimodal frames from a simulated fully mechanized mining face show 98.5% mAP@0.5 for tri-modal detection, an approximately 10 Hz integrated refresh rate, a 3D keypoint localization MAE of 1.40 cm, and a maximum environmental-condition clearance error of 2.2 cm under combined dust-and-mist disturbance. These results demonstrate the proposed method has potential application value in the field of intelligent mines.

## 1. Introduction

Hydraulic-support face guards, shearer drums, and ranging arms operate in a tightly coupled spatial envelope in fully mechanized coal mining faces. When the face guard does not retract or align in time, the shearer cutting mechanism may intrude into the guard space and trigger cutting interference, which can damage cutting picks, drums, guard plates, and support structures, interrupt production, and increase the risk of unsafe local support [1]. The warning problem is therefore not only an object-recognition problem. A useful monitoring system must estimate the relative pose of the face guard, reconstruct a geometric envelope that can be interpreted by the control system, and convert the minimum clearance to a warning state quickly enough for active intervention.

Reliable warning is difficult because fully mechanized faces are narrow, dusty, misty, strongly vibrating, and weakly illuminated. Visible imagery provides fine texture, straight edges, and local shape information, but image contrast decreases sharply under low illumination and suspended coal dust. Infrared imaging is less dependent on visible illumination and can preserve the thermal saliency of mechanical components under weak light, but its spatial resolution and boundary sharpness are lower than those of visible imagery. LiDAR provides direct metric geometry and is attractive for distance calculation, yet airborne dust and water droplets can attenuate laser returns, create isolated outliers, and reduce the completeness of target surfaces [2,3,4,5,6]. These complementary strengths and failure modes motivate a multi-source perception architecture rather than reliance on a single sensing route.

Large-scale mobile-perception benchmarks and adverse-weather studies have shown that multimodal datasets and camera–LiDAR fusion are important for robust 3D perception when semantics and geometry are distributed across different sensors [7,8,9,10]. However, a fully mechanized mining face differs from open-road scenes in three important ways. First, the target is a hydraulic-support component with repeated metal surfaces and partial occlusion rather than a free-standing traffic object. Second, the clearance that determines cutting interference is often on the order of centimeters, so the perception output must preserve metric reliability. Third, the environment contains dust, water mist, strong local vibration, and limited installation space, which makes standard camera–LiDAR fusion pipelines difficult to apply directly.

The proposed multi-source system addresses this gap by combining RGB texture, infrared saliency, and LiDAR geometry in a common spatial frame. The system first improves the quality of each modality, then synchronizes and registers the data streams, extracts multi-scale visual and geometric features, and adaptively weights the three modalities according to their frame-level quality. The fused representation is used for face-guard detection, corner keypoint localization, 6DoF pose solving, and minimum-distance warning. Compared with a pure image classifier, the output is physically interpretable; compared with a single point-cloud detector, the image and thermal branches provide semantic constraints when the point cloud is incomplete. This paper therefore explores a multimodal fusion route to the same mining-safety problem and focuses on pose-level warning rather than point-level target segmentation alone.

This work evaluates the sensor combination and the fusion architecture separately. The sensor combination supplies complementary observations, whereas the architecture contributes (i) a common LiDAR-centered BEV reference, (ii) frame-level modality-quality weighting, (iii) axial cross-modal aggregation, and (iv) pose- and clearance-level outputs. To evaluate the contribution of each modality and fusion module, this study conducts modality ablations, fusion-module ablations, and determines early- and late-fusion baselines and safety-oriented metrics.

The main contributions are as follows:

1. A synchronized RGB/infrared/LiDAR dataset and annotation workflow for face-guard detection and pose estimation, built from 2400 frames collected under representative mining-face conditions.
2. A multi-source visual fusion pipeline that integrates image denoising, temporal synchronization, spatial registration, Swin-T feature extraction, BEV-aligned LiDAR encoding, and adaptive cross-modal weighting.
3. A face-guard pose and interference-warning method that combines 2D detection, corner keypoint recovery, EPnP pose solving, and 3D envelope distance evaluation.

## 2. Materials and Methods

### 2.1. System Overview

Figure 1 summarizes the proposed pipeline. RGB, infrared, and LiDAR streams are first cleaned and synchronized, then aligned into a common LiDAR-centered coordinate frame. Multi-scale features are extracted from each modality, fused adaptively, and passed to a face-guard detection head. The predicted 2D box and keypoints are lifted to 3D pose by geometric constraints and refined with temporal filtering before the minimum distance to the shearer drum is mapped to a warning state.

The workflow was designed around the engineering sequence of cutting-interference monitoring. The acquisition layer records visible texture, infrared thermal contrast, and 3D point-cloud geometry from a co-mounted sensing unit. The preprocessing layer removes modality-specific noise and converts all inputs to calibrated measurements. The registration layer establishes a unified temporal and spatial reference, because a small timestamp offset or extrinsic error can create a false clearance change when the face guard or the shearer arm is moving. The feature layer extracts texture, thermal, and geometric descriptors, and the fusion layer combines these descriptors in a BEV-centered representation. Finally, the decision layer converts perception results into a minimum distance and a discrete warning state. This layered design makes the method auditable: each processing stage has a clear physical role and can be checked independently during deployment.

For one synchronized frame *t*, the input is denoted as

(1) $$X_{t}=\left\{I_{t}^{r},I_{t}^{i},P_{t}^{l}\right\},$$

where $I_{t}^{r}$ is the RGB image, $I_{t}^{i}$ is the infrared image, and $P_{t}^{l}={\left\{p_{n}\right\}}_{n=1}^{N_{t}}$ is the LiDAR point cloud in the LiDAR coordinate frame. The final output is

(2) Yt={b^t,K^t,R^t,t^t,Dt,st},

including the 2D face-guard bounding box b^t, the projected keypoints K^t, the estimated rotation and translation (R^t,t^t), the minimum clearance $D_{t}$, and the warning state $s_{t}$.

### 2.2. Experimental Platform and Sensors

The experiment was conducted on a 1:1 simulated fully mechanized mining-face platform. The physical platform includes a cutting drum mock-up, a ranging arm, a hydraulic-support face guard, a dual-channel RGB/infrared camera, a 32-line LiDAR, and an edge computer for data acquisition and processing. Figure 2 shows the platform layout, and Figure 3 shows the sensing assembly.

The dual-channel camera was the KBA12R mining intrinsically safe thermal-imaging camera. Its visible channel provided 8-bit RGB images with a nominal resolution of 1920 × 1080, at least 400 TVL horizontal clarity, and a 0.05 lux low-light response. Its infrared channel used a VOx uncooled focal-plane array with 640 × 512 resolution, NETD below 50 mK, 16-bit raw output, and 25 fps acquisition. The 32-line LiDAR was identified as a RoboSense RS-LiDAR-32 [11]. Its published specifications include a 360° horizontal field of view, an approximately −25° to +15° vertical field of view, an approximate minimum vertical angular resolution of 0.33°, a nominal maximum range of 200 m under the manufacturer’s stated reflectivity condition, and selectable 5, 10, and 20 Hz scan rates.

The platform was built to reproduce the local spatial relationship that causes cutting interference. The cutting drum and ranging arm mock-up represent the dangerous region of the shearer, while the hydraulic-support face guard is mounted on an adjustable mechanism to generate representative guard poses. The sensing unit is fixed near the simulated ranging-arm position so that the target appears in a constrained field of view similar to a practical installation. Normal, dust, mist, and combined dust–mist environments were reproduced to evaluate whether modality degradation changes the distance output. Because the same mechanical platform is used for all modality combinations, the ablation results reflect sensing and algorithmic differences rather than different geometric conditions.

Table 1 summarizes the key sensor specifications. The two camera channels were co-aligned in the sensing assembly, and the LiDAR was mounted on the same rigid bracket near the simulated ranging-arm position. The global mechanical frame was fixed at the geometric center of the shearer base, with the x-axis along the traction direction and the z-axis normal to the platform floor.

The three sensors were selected to cover different information channels (Table 2). The RGB channel contributes high-frequency texture and edge details that are useful for 2D bounding boxes and corner heatmaps. The infrared channel contributes thermal contrast and therefore stabilizes the target foreground when visible-light contrast is reduced by weak illumination or dust scattering. The LiDAR channel contributes direct 3D coordinates and is the primary source for metric distance estimation. The edge computer runs acquisition, synchronization, inference, and warning-state output; in the reported implementation, the integrated loop is refreshed at approximately 10 Hz. The training experiments used a single NVIDIA RTX 3090 GPU, whereas the integrated inference verification used an NVIDIA GeForce RTX 5080 Laptop GPU with 16 GB memory, an Intel Core Ultra 9 275HX CPU, and 32 GB RAM. The integrated refresh rate was approximately 10 Hz under laboratory conditions. Mine-specific controller timing requirements still require field validation.

### 2.3. Dataset Construction and Annotation

The dataset contains 2400 synchronized multimodal frames collected from the simulated mining-face platform (Table 3). For model development, the frames were randomly divided at the frame level into 1680 training frames, 480 validation frames, and 240 held-out frame-level test frames. To evaluate the stability of the frame-level results against sample selection, we additionally performed repeated frame-level resampling/cross-validation over 360 combinations. Because these experiments were based on frame-level sampling from the same laboratory platform, the results are interpreted as frame-level generalization. In addition, a manually selected 400-frame warning-evaluation set was used for the four-level warning analysis. The frames were selected to reduce the influence of adjacent samples from the same continuous action. This 400-frame set is used only for warning-performance evaluation and is not treated as an independent sequence-level test set. All samples were annotated with SUSTechPOINTS, including 2D face-guard boxes, corner keypoints, and the associated 3D geometry needed for pose recovery.

Experimental condition design for the multimodal dataset is shown in Table 4. The data were collected to cover both pose variation and sensing disturbance. The face guard was adjusted over the effective working range, from retracted to near fully extended states, while the simulated ranging arm was placed at low, middle, and high pitch states. Environmental disturbance was introduced as four categories: normal visibility, light dust, light mist, and combined dust–mist disturbance. After removing blurred frames, incomplete synchronization records, and frames in which the face guard was outside the effective field of view, the remaining frames were balanced into six standard groups of 400 frames. Each frame group contains paired RGB, infrared, and LiDAR observations, so modality ablations use identical target states.

Annotation was performed in a joint image–point-cloud view. The RGB and infrared images were labeled with the face-guard 2D bounding box and four visible corner keypoints. The LiDAR point cloud was used to check the spatial consistency of the labeled target and to provide the corresponding 3D guard-plate geometry for pose solving. Frames close to the warning thresholds were inspected with higher priority because a small keypoint or registration error can change the discrete warning state. The dataset therefore supports three levels of evaluation: detection quality, pose and distance accuracy, and final warning classification.

### 2.4. Preprocessing and Spatiotemporal Registration

Image preprocessing included denoising and lens-distortion correction. LiDAR preprocessing included invalid-point removal, outlier suppression, and voxel-style downsampling to reduce sparse clutter. Time synchronization used a hardware trigger centered on the LiDAR timing reference, followed by Kalman-style delay compensation to reduce residual offsets to the millisecond level. Spatial alignment used coarse registration from natural-scene features, hand–eye calibration, and NDT-ICP refinement. Camera geometry followed the standard pinhole projection model [13], while rigid alignment and refinement were supported by RANSAC, closed-form point-set alignment, hand–eye calibration, and ICP [14,15,16,17,18].

#### 2.4.1. Image Denoising and Distortion Correction

Visible and infrared images in the mining-face scene contain three common degradations: impulse noise caused by light scattering on dust particles, Gaussian-like sensor noise under weak illumination, and stripe-like interference from electric and hydraulic equipment. A two-stage denoising process was therefore used. The first stage uses a trimmed mean filter to suppress impulse noise:

(3) $$\overset{˜}{I}\left(u\right)=\frac{1}{|\Omega |-2q}\sum_{v\in \Omega_{u}\setminus E_{q}}I\left(v\right),$$

where $\Omega_{u}$ is the local window centered at pixel u and $E_{q}$ contains the *q* largest and *q* smallest gray values in the window. This operation removes isolated bright and dark points without replacing the center pixel by a hard median in all cases.

The second stage applies multi-scale bilateral filtering. For a scale *s*, the bilateral output is

(4) $$I_{b}^{s}\left(u\right)=\frac{1}{W_{u}}\sum_{v\in \Omega_{u}^{s}}G_{\sigma_{s}}(∥u-v∥)G_{\sigma_{r}}(|I\left(u\right)-I\left(v\right)|)I\left(v\right),$$

where $G_{\sigma_{s}}$ and $G_{\sigma_{r}}$ denote spatial and range kernels. Outputs from 3×3, 5×5, and 7×7 windows are weighted according to the local Sobel gradient magnitude, so edge regions retain more small-window information and flat regions receive stronger smoothing. Lens distortion is then corrected with a radial–tangential model:

(5) $$\begin{array}{cc} x_{d} & =\;\;x\left(1+k_{1}r^{2}+k_{2}r^{4}\right)+2p_{1}xy+p_{2}\left(r^{2}+2x^{2}\right),\\ y_{d} & =\;\;y\left(1+k_{1}r^{2}+k_{2}r^{4}\right)+p_{1}\left(r^{2}+2y^{2}\right)+2p_{2}xy, \end{array}$$

where (x,y) and $\left(x_{d},y_{d}\right)$ are normalized undistorted and distorted coordinates. Natural straight edges from the face guard, support frame, and roadway structure were used as calibration cues when an artificial target could not be installed conveniently.

#### 2.4.2. LiDAR Point-Cloud Filtering

The raw LiDAR point cloud was processed to reduce dust-related outliers and redundant points before registration and BEV encoding. Points with NaN coordinates, abnormal intensity values, and ranges outside the useful working volume were removed first. For a point $p_{i}$, the range filter is

(6) $$r_{\min}\leq {∥p_{i}∥}_{2}\leq r_{\max}.$$

Statistical outlier removal was then applied. For each point, the mean distance to its *k* nearest neighbors is

(7) $${\overset{¯}{d}}_{i}=\frac{1}{k}\sum_{p_{j}\in N_{k}\left(p_{i}\right)}{∥p_{i}-p_{j}∥}_{2}.$$

The point is rejected if ${\overset{¯}{d}}_{i}>\mu_{d}+\lambda \sigma_{d}$, where $\mu_{d}$ and $\sigma_{d}$ are the mean and standard deviation of neighbor distances in the frame. Finally, voxel-grid downsampling replaces points in each voxel with their centroid:

(8) $$c_{v}=\frac{1}{|V_{v}|}\sum_{p_{i}\in V_{v}}p_{i}.$$

This step reduces computation while preserving the face-guard plane and the spatial envelope of the shearer mechanism. The statistical neighborhood size was set to k=15, and points were rejected when ${\overset{¯}{d}}_{i}>\mu_{d}+2\sigma_{d}$. The voxel size used in the implementation was 0.08 m.

#### 2.4.3. Temporal Synchronization

The LiDAR scan clock was used as the global timing reference. A hardware trigger provides the first alignment between the LiDAR, RGB, and infrared streams. Residual delay is modeled as part of a state vector:

(9) $$x_{t}={\left[q_{t}^{T},{\dot{q}}_{t}^{T},\Delta t_{r,t},\Delta t_{i,t}\right]}^{T},$$

where $q_{t}$ and ${\dot{q}}_{t}$ are the target position and velocity, and $\Delta t_{r,t}$ and $\Delta t_{i,t}$ are the residual RGB and infrared delays relative to the LiDAR reference. A Kalman-style predictor compensates these delays before feature-level alignment. This compensation is important because a 50 ms offset can create a 5 cm apparent position error when equipment moves at 1 m/s, which is comparable to the lowest warning threshold in the proposed system.

The filtering protocol used a constant-velocity state at the 10 Hz update rate, with the residual RGB and infrared delays modeled as random-walk states. The implementation uses an initial position covariance of 0.05^2^ m^2^, velocity covariance of 0.20^2^ m^2^/s^2^, delay covariance of 0.5^2^ ms^2^, and measurement noise of 0.01^2^ m^2^ for the position observation.

#### 2.4.4. Spatial Registration

Spatial registration was performed in three stages. First, natural straight lines and planes in the images and LiDAR point cloud were extracted by Canny/Hough transform and RANSAC plane fitting, respectively, to obtain an initial rigid transformation. Second, hand–eye calibration used controlled face-guard or support motion to refine the relative pose between the sensing assembly and the mechanical coordinate frame. Third, NDT-ICP refinement minimized local distribution mismatch between the transformed point cloud and reference geometry. The registration objective can be written as

(10) (R^,t^)=argminR,t∑nρRpnl+t,Mc,

where $\rho (\cdot ,M_{c})$ is the residual to the camera-derived or reference geometric model. After registration, all modalities are represented in a LiDAR-centered coordinate frame for BEV fusion and distance calculation.

For a LiDAR point $p_{l}={\left[x_{l},y_{l},z_{l}\right]}^{T}$ and the camera extrinsics $\left(R_{lc},t_{lc}\right)$, the camera-frame point is

(11) $$p_{c}=R_{lc}p_{l}+t_{lc},$$

and the image projection is

(12) $$u=f_{x}\frac{x_{c}}{z_{c}}+c_{x},\;v=f_{y}\frac{y_{c}}{z_{c}}+c_{y}.$$

This alignment makes image features and LiDAR BEV features comparable in a single coordinate frame.

### 2.5. Multimodal Feature Extraction and Fusion

The RGB and infrared branches use Swin-T backbones to extract multi-scale features, followed by feature pyramid aggregation [19,20]. The LiDAR branch converts point clouds into BEV-aligned geometric features, following the efficiency principle of sparse point-cloud encoders and BEV detectors [21,22,23,24,25]. Image features are sampled into the LiDAR BEV frame by bilinear interpolation at projected reference points, so the three modalities share the same spatial basis.

For the RGB and infrared branches, shallow layers preserve local edge information while deeper layers encode larger-scale context. The feature pyramid combines these levels as

(13) $$F_{\mathrm{pyr}}^{m}=A\left(F_{1}^{m},F_{2}^{m},F_{3}^{m},F_{4}^{m}\right),\;m\in \left\{r,i\right\},$$

where A(·) denotes lateral projection, upsampling, and concatenation. This design is useful for the face guard because its outer contour may be only a few pixels wide in the infrared image but occupies a larger geometric region in the LiDAR frame. The LiDAR point cloud is voxelized within the region of interest and projected to a BEV grid. Each occupied cell stores height, point density, and aggregated intensity or return statistics, which are processed by a sparse encoder to produce $F_{\mathrm{BEV}}^{l}$.

The image-to-BEV transformation uses the calibrated projection. For a BEV reference cell b, its corresponding camera coordinates are obtained from the inverse rigid transform and sampled with bilinear interpolation:

(14) $$F_{m\to \mathrm{BEV}}\left(b\right)=\sum_{u\in N(\pi_{m}\left(b\right))}w_{u}F_{\mathrm{pyr}}^{m}\left(u\right),$$

where $\pi_{m}$ is the projection from the unified frame to modality *m*, N(·) is the four-pixel interpolation neighborhood, and $w_{u}$ represents the normalized interpolation weights. Points projected outside the valid image area are masked rather than extrapolated, preventing invalid image features from contaminating the LiDAR representation.

The BEV region of interest was discretized at 0.10 m resolution in the horizontal plane. Each occupied pillar retained height statistics, point count, and mean return intensity. The input images were resized to 960 × 540 for training and inference, while the original sensor streams were retained for calibration and visualization. Random horizontal translation, RGB brightness perturbation, infrared-intensity perturbation, and random point dropout were used as training augmentations, and geometric labels were transformed together with the corresponding modality.

The fusion design follows two principles established in camera–LiDAR 3D perception: geometric features should remain metric in the BEV frame, while image features should contribute semantic and boundary evidence at projected positions [26,27,28,29,30,31]. In the mining-face scene, this alignment is especially important because the visible target boundary may be weakened by dust or low illumination, whereas the infrared branch still captures thermal contrast and the LiDAR branch keeps the 3D guard/drum geometry.

Let $F_{r}$, $F_{i}$, and $F_{l}$ denote the RGB, infrared, and LiDAR feature maps. The adaptive fusion block produces

(15) $$F_{\mathrm{fuse}}=\alpha_{r}F_{r}+\alpha_{i}F_{i}+\alpha_{l}F_{l},$$

with

(16) $$\alpha_{m}=\frac{\exp(s_{m})}{\sum_{k\in \{r,i,l\}}\exp\left(s_{k}\right)},$$

where $s_{m}$ is a modality-quality score derived from image contrast, thermal saliency, and LiDAR point density. The weighting therefore suppresses a degraded stream rather than forcing equal contribution from all sensors. An axial cross-modal fusion layer then models long-range dependencies among the aligned multimodal features, producing a fused representation that preserves visible edges, thermal saliency, and metric depth cues in the same BEV coordinate frame.

The quality scores are computed from frame-level statistics rather than fixed modality coefficients. For example, visible-image contrast is represented by the normalized standard deviation and gradient energy, infrared saliency is represented by the contrast between the target region and its local background, and LiDAR quality is represented by valid-point density and the fraction of points surviving outlier removal. The score for modality *m* is written as

(17) $$s_{m}=w_{m}^{T}z_{m}+b_{m},$$

where $z_{m}$ is the normalized quality vector and $\left(w_{m},b_{m}\right)$ are learned parameters. The softmax normalization in Equation (16) makes the weights comparable across frames. Consequently, a degraded RGB stream can be down-weighted while infrared and LiDAR features retain their contribution.

After quality weighting, an axial cross-modal fusion block performs attention along the horizontal and vertical BEV axes. In contrast with a fully dense two-dimensional attention operation, axial attention reduces the computational cost while retaining long-range dependencies between the guard boundary and the shearer dangerous region. The fused output is

(18) $$F_{\mathrm{out}}=C\left(F_{\mathrm{fuse}},A_{h}\left(F_{\mathrm{fuse}}\right),A_{v}\left(F_{\mathrm{fuse}}\right)\right),$$

where $A_{h}$ and $A_{v}$ denote horizontal and vertical attention and C is a channel projection with residual connection. The output retains the metric BEV coordinate system, which is essential for the subsequent distance calculation.

The RGB and infrared Swin-T branches used a 4 × 4 patch size, a base embedding dimension of 96, and a window size of 7. The axial block used 8 attention heads at the fused feature scale and a residual channel projection. The modality-quality vector was z-score-normalized using training-set statistics separately for RGB contrast/gradient energy, infrared target-background contrast, and LiDAR valid-point density/retention ratio.

### 2.6. Guard-Plate Detection and 6DoF Pose Estimation

The fused features feed a detection head that outputs the face-guard bounding box and four corner keypoints. The detector follows a dense one-stage design with FPN-style multi-scale features and FCOS-like classification and regression branches [32]. Focal loss is used to reduce the impact of the heavy background-to-target imbalance [33].

The detection head has three coupled outputs. The classification branch predicts the probability that a BEV-aligned feature location belongs to the face guard, the regression branch predicts the 2D bounding-box offsets, and the keypoint branch predicts four corner heatmaps and their local offsets. The total training objective is

(19) $$L=\lambda_{\mathrm{cls}}L_{\mathrm{focal}}+\lambda_{\mathrm{box}}L_{\mathrm{box}}+\lambda_{\mathrm{kp}}L_{\mathrm{kp}}+\lambda_{\mathrm{pose}}L_{\mathrm{reproj}},$$

where the four terms represent classification, box regression, keypoint localization, and pose reprojection consistency. The box term combines an overlap loss with an $\ell_{1}$ regression term. The keypoint term is computed on the four annotated corners and uses a robust penalty to reduce the influence of partially occluded corners. The reprojection term is introduced after an initial pose is obtained and encourages the estimated pose to project back to the observed keypoints.

The training configuration used AdamW with an initial learning rate of $1\times 10^{-4}$, weight decay $5\times 10^{-4}$, cosine learning-rate decay, 120 maximum epochs, batch size 16, and a five-epoch linear warm-up from $1\times 10^{-6}$. Early stopping was applied after 15 validation epochs without mAP improvement. The loss weights were $\lambda_{\mathrm{cls}}=1.0$, $\lambda_{\mathrm{box}}=1.0$, $\lambda_{\mathrm{kp}}=2.0$, and $\lambda_{\mathrm{pose}}=1.5$.

Given the predicted 2D keypoints u^j and corresponding 3D guard-plate points $X_{j}$, the pose is estimated by

(20) θ^=argminθ∑j=14π(θ,Xj)−u^j22,

where θ=(R,t) and π(·) is the camera projection model. EPnP provides the initial solution, Levenberg–Marquardt refinement reduces reprojection error, and an adaptive Kalman filter stabilizes the final 6DoF trajectory [34].

The four 3D keypoints are defined in a guard-plate coordinate system whose origin is located at the plate center, with the local *x*-axis along the plate width and the local *y*-axis along its height. The rotation matrix is parameterized by a Rodrigues vector during nonlinear refinement. The reprojection residual is

(21) ej=π(RXj+t)−u^j,

and the refined pose minimizes $\sum_{j}\rho (∥e_{j}{∥}_{2})$ with a robust Huber penalty ρ. Frames with an insufficient number of valid LiDAR associations use the image keypoints for initialization and retain the previous valid pose as a temporal prior. A Kalman-style update then limits frame-to-frame displacement and reduces small threshold-crossing oscillations.

### 2.7. Distance Model and Four-Level Warning

The recovered guard-plate pose is converted to a 3D envelope $E_{g}$, and the shearer drum is represented by a second envelope $E_{d}$. The minimum interference distance is

(22) $$D_{t}=\min_{x\in E_{g},y\in E_{d}}{∥x-y∥}_{2}.$$

The guard envelope is constructed by transforming the four corner points and the plate thickness limits into the unified frame. The shearer dangerous envelope contains the cutting drum cylinder and the portion of the ranging arm that can enter the guard region. If the drum center is $c_{d}$, its nominal radius is $r_{d}$, its width is $w_{d}$, and the unit axis is $a_{d}$, a sampled drum surface point can be written as

(23) $$y\left(\phi ,\ell \right)=c_{d}+\ell a_{d}+r_{d}\left(e_{1}\cos\phi +e_{2}\sin\phi \right),$$

where $\ell \in [-w_{d}/2,w_{d}/2]$, ϕ∈[0,2π), and $\left(e_{1},e_{2},a_{d}\right)$ is an orthonormal basis. A small mechanical safety margin is added to the nominal drum radius to account for cutting picks and vibration. The minimum distance is evaluated between the guard envelope and the sampled dangerous surfaces, so the output corresponds to a conservative clearance rather than the distance between two centroids.

To make the decision robust to sensor noise, the distance output is filtered before thresholding. Let ${\overset{˜}{D}}_{t}$ denote the exponentially smoothed distance and let η be the smoothing coefficient:

(24) $${\overset{˜}{D}}_{t}=\eta D_{t}+\left(1-\eta \right){\overset{˜}{D}}_{t-1}.$$

The warning state is changed only after consecutive-frame confirmation and uses a small hysteresis interval around each threshold. This prevents a single noisy frame from causing an unnecessary emergency transition while preserving the conservative behavior required near the dangerous levels. In the implementation, the hysteresis half-width was 20 mm and a new state required confirmation in three consecutive 10 Hz frames. The thresholds were set through multiple tests and were defined as follows: 50 mm is the minimum laboratory clearance at which the expanded drum/guard envelopes approach the collision boundary; 150 mm is the trend-warning boundary that allows speed reduction and posture adjustment; and 300 mm is the attention boundary at which a persistent clearance reduction should be monitored.

The 10 Hz update rate corresponds to an interval of approximately 100 ms. Requiring three consecutive frames to confirm a new warning state adds approximately 0.2–0.3 s of confirmation delay, depending on the event phase relative to the sampling instant. Including the measured integrated processing latency of approximately 96 ms, the laboratory perception-and-decision delay is approximately 0.30–0.40 s before external communication, controller execution, and mechanical or hydraulic actuation are considered. For equipment speed *v*, the additional travel distance during the total response time is $d_{\mathrm{delay}}=v\;\Delta t_{\mathrm{total}}$, where $\Delta t_{\mathrm{total}}$ includes sensing, computation, communication, controller, and actuation delays. The system uses four warning levels:

(25) $$s_{t}=\left\{\begin{array}{cc} L0, & D_{t}>300\;\mathrm{mm},\\ L1, & 150\;\mathrm{mm}<D_{t}\leq 300\;\mathrm{mm},\\ L2, & 50\;\mathrm{mm}<D_{t}\leq 150\;\mathrm{mm},\\ L3, & D_{t}\leq 50\;\mathrm{mm}. \end{array}\right.$$

The control loop refreshes at 10 Hz and uses a ±20 mm hysteresis band with three-frame confirmation to suppress threshold jitter. The warning definition is presented more clearly in Table 5.

### 2.8. Evaluation Metrics

Detection performance is reported using mAP@0.5 and mAP@0.5:0.95. Pose quality is reported using corner-point localization error, reprojection error, translation error, and rotation error. System-level reliability is evaluated with the four-level classification accuracy, precision, recall, F1-score, dangerous-level recall, and the runtime of the integrated warning loop.

For a set of *N* annotated keypoints, the mean keypoint error is

(26) Ekp=1N∑j=1NX^j−Xj2,

where X^j and $X_{j}$ are the estimated and reference 3D positions. For a measured distance D^n and a reference distance $D_{n}$, the absolute and relative errors are

(27) en=|D^n−Dn|,enrel=|D^n−Dn|Dn×100%.

The mean absolute error and root mean square error are $\mathrm{MAE}=N^{-1}\sum_{n}e_{n}$ and RMSE=N−1∑n(D^n−Dn)2. For pose validation, the translation error is et=∥t^−t*∥2 and the rotation error is eR=cos−1((tr(R^R*T)−1)/2), where $\left(R^{*},t^{*}\right)$ is the platform-derived ground truth. The overall warning accuracy is the ratio of correctly classified frames to the total number of frames in the additional 400-frame warning-evaluation set. For safety reporting, dangerous-level recall is defined for L2 and L3 jointly as the fraction of reference L2/L3 frames not assigned to L0, and ‘no missed detections’ is defined as no reference L2/L3 frame being classified as L0 in this study. It does not require every dangerous frame to be assigned to its exact original level.

## 3. Results

### 3.1. Fusion Detection Performance

Table 6 reports the detection results of different modality combinations. Table 7 reports the held-out frame-level architecture comparison. RGB-only detection reached 89.2% mAP@0.5, infrared-only detection reached 83.6%, and RGB + IR fusion reached 92.4%. Adding LiDAR increased the pooled tri-modal result to 98.5% mAP@0.5 and an approximately 10 Hz integrated refresh rate.

The pooled single-modality diagnostics show different failure modes. RGB detection was stable in normal and well-lit frames, but confidence decreased when dust reduced texture contrast and blurred the face-guard edge. Infrared detection remained more stable under weak illumination, but the lower spatial resolution and thermal boundary diffusion reduced box precision and keypoint sharpness. The RGB +infrared result improved by 3.2 percentage points over RGB-only detection because thermal saliency compensated for visible-image degradation. However, image-only fusion still lacks direct metric depth, so corner recovery remains sensitive when the guard surface and background have similar visual appearance. Adding LiDAR increased the pooled mAP@0.5 to 98.5% because BEV geometry constrains the target location in depth and stabilizes the fused feature around the physical guard boundary. These observations are interpreted as modality diagnostics; the held-out frame-level architecture comparison is given in Table 7.

To distinguish the sensor contribution from the architecture contribution, representative baselines were evaluated under the same held-out frame-level test-set protocol. The comparison results are shown in Table 8.

The comparison suggests that the proposed architecture contributes beyond simply adding a third sensor: adaptive weighting and axial aggregation improve the held-out frame-level scores while retaining a lower latency than the early- and late-fusion alternatives.

Figure 4 shows representative qualitative outputs under two face-guard poses. The fused system preserves detection stability across different target orientations and background structures.

Qualitatively, the fused outputs retain the face-guard outline even when one modality becomes ambiguous. In frames with dust, the visible-light edge is weakened, but infrared thermal contrast still indicates the target region and LiDAR points provide the 3D boundary. In frames where the LiDAR surface is locally sparse, the image branches still constrain the 2D contour and prevent the detector from extending the guard region into background metal structures. This complementary behavior explains why the gain from LiDAR is reflected not only in the mAP but also in the subsequent distance output.

To present the advantages of the multi-source fusion method objectively while acknowledging failure modes under severe interference, we discuss representative stress cases. Figure 5 collects qualitative cases observed during the laboratory experiments. Sub-figure (a) shows reduced visible-light contrast, while subfigures (b) and (c) show sparse returns and a changed guard orientation. The intended fallback is to reduce the quality weight of the degraded stream, initialize the pose from valid keypoints and the previous state, and retain the geometric envelope only when the registration residual remains within its acceptance limit. These mechanisms provide conservative fallback behavior under severe laboratory disturbances, but field validation is still required. The main technical difficulties and the proposed methods to mitigate them are summarized in Table 9.

### 3.2. Pose Recovery and Distance Accuracy

Figure 6 shows the point-cloud pose sequence across representative face-guard angles. The recovered geometry remains consistent across pose changes, indicating that the fusion-based implementation is stable enough for distance estimation.

The complete 6DoF pose was validated against a mechanical reference rather than using keypoint error alone (Table 10). The face-guard fixture was first measured in the platform coordinate system, and each test pose was defined by the calibrated extension angle, ranging-arm pose, and reference clearance. Four plate corners were then transformed into the camera frame using the calibrated rigid transform. The reference translation was checked with a contact/laser range measurement, and the reference rotation was obtained from the fixture angle marks and the calibrated plate normal.

The protocol above separates the pose target from the downstream clearance target: translation and rotation quantify the recovered rigid pose, while reprojection and 3D keypoint errors diagnose the image-to-geometry link.

To further evaluate the distance output, 20 representative distances were measured with a reference laser rangefinder in the laboratory calibration setting. The model of laser distance meter used was A10Apex. Its effective measurement range is 50 m and the measurement accuracy is ±2 mm. This distance measuring instrument was installed and fixed by a bracket, enabling it to detect the distance of the target from various angles. The selected values covered near-threshold regions around 5 cm, 15 cm, and 30 cm, because errors in these ranges are most likely to change the warning level. Table 11 shows that the absolute error of the sampled distances was between 0.1 and 0.5 cm. In the 20 rows, the descriptive MAE, RMSE, bias, sample standard deviation, and maximum absolute error are 0.28 cm, 0.32 cm, −0.05 cm, 0.32 cm, and 0.50 cm, respectively. This 0.28 cm MAE describes the complete calibrated clearance-estimation pipeline in the clean, short-range laboratory experiment; it is not the absolute accuracy of a single LiDAR return. RGB/IR keypoints constrain the guard pose, multiple LiDAR returns provide metric geometry, and outlier rejection, envelope fitting, and temporal smoothing reduce the influence of isolated point-level noise. Systematic calibration error, sensor accuracy, and environmental disturbance remain relevant.

As shown in Table 12, the maximum errors in the distance-related tables refer to different evaluation settings. The 20-point clean calibration experiment had a maximum absolute error of 0.50 cm. The environmental-condition test had a maximum error of 2.20 cm under combined dust and mist. The threshold-neighborhood diagnostic reported raw pre-filter distance errors and had a maximum value of 2.80 cm near the 300 mm threshold. A raw label change in Table 13 means that the unfiltered distance crossed the threshold relative to the reference label. The hysteresis and three-frame confirmation logic were applied after this diagnostic, so the table indicates the sensitive pre-filter case.

### 3.3. Fusion Effect and Warning Classification

To evaluate the four-level warning classification, we manually selected an additional 400-frame warning-evaluation set from the synchronized data. The selection reduced the influence of adjacent samples from the same continuous action. The set contained 200, 100, 60, and 40 reference frames for L0, L1, L2, and L3, respectively. The complete matrix and class-wise metrics are reported in Table 14 and Table 15.

Taking into account the actual engineering requirements, in this study, “no missed detections” means that no reference L2 or L3 frame is assigned to L0; it does not mean that every dangerous frame is assigned to its exact original level. In this experiment, the joint L2/L3 safety recall is 100.00%, while the exact-level recall for L2 and L3 is 96.00%. The distinction is reported explicitly because a conservative adjacent-level warning is less severe than a missed dangerous event.

The fusion effect is reflected at both the perception and decision levels. In the pooled modality diagnostic, RGB + IR fusion improved mAP@0.5 by 3.2 percentage points over RGB-only detection, and adding LiDAR improved it by a further 6.1 percentage points over RGB + IR.

## 4. Discussion

The results show that the face-guard warning problem is not solved by detection alone. The main failure mode in underground mines is modality collapse: visible images fail in dust and low light, infrared images lack fine geometry, and LiDAR alone cannot supply enough semantics. The tri-modal fusion strategy addresses this by letting each modality cover the weakness of the others. The 9.3-point gain over RGB-only detection and the 6.1-point gain over RGB + IR fusion are consistent with this interpretation.

The pose and distance results are more important than the detection score because the control decision depends on spatial clearance. The centimeter-level pose error and 2.8 cm raw pre-filter threshold-neighborhood error remain below the 50 mm interference threshold in the laboratory diagnostic, but this margin should not be interpreted as an equipment-level stopping-distance guarantee. In addition, the four-level policy separates early attention from true collision risk, which is more useful in a working face than a binary safe/unsafe decision.

The proposed approach is also distinct from a 4D millimeter-wave radar route to the same mining-safety problem. Both routes can be tested on the same experimental platform because the physical interference mechanism is identical: the warning decision always depends on the relative spatial relationship between the shearer dangerous region and the face guard. However, the sensing principles and algorithmic outputs are different. A radar-only method emphasizes sparse radar point-cloud preprocessing, semantic segmentation of target points, and bounding-box fitting. The present study emphasizes heterogeneous visual fusion, 2D/3D keypoint localization, 6DoF pose estimation, and four-level warning from a reconstructed geometric envelope. The common platform is useful because it allows different sensing routes to be compared under consistent mechanical geometry.

The advantage of the multi-source visual route is that the recovered pose carries richer geometric interpretation than a target label or a point cluster. The RGB and infrared branches preserve target appearance and boundary saliency, while LiDAR constrains the metric depth. This makes the method suitable for a control-oriented warning variable. The limitation is that the system requires more calibration effort than a single-sensor system. It depends on stable extrinsics, time synchronization, and the continued availability of at least two useful modalities. A radar route may be more robust to optical visibility collapse, whereas the proposed visual–LiDAR fusion route provides denser semantic and pose information when calibration is maintained.

The current study is still limited to a simulated fully mechanized face and a 2400-frame dataset. Field deployment will require more backgrounds, more motion patterns, stronger vibration, and longer-term drift tests. The present experiments do not yet cover all coupled motions among shearer traction, drum rotation, ranging-arm height adjustment, hydraulic-support action, and moving coal flow. The warning thresholds were selected from equipment clearance requirements and laboratory calibration, but mine-specific threshold tuning will be necessary before operational deployment. In addition, the current implementation was evaluated on an edge computing unit in a controlled setting; future work should test embedded deployment, sensor protection, calibration drift, controller communication delay, actuator response, stopping distance, and fault-handling logic during longer operating periods.

Even with those limitations, this study demonstrates a sensing method with potential competitiveness for underground warning: multimodal acquisition, spatiotemporal alignment, adaptive fusion, 6DoF pose recovery, distance estimation, and warning-state output. The results indicate that a tri-modal system can reduce the risk of single-modality failure and provide a physically meaningful clearance variable for active monitoring. This makes the method a useful step toward intelligent coordination between shearers and hydraulic supports in fully mechanized coal mining faces.

## 5. Conclusions

This paper presents a multi-source visual fusion system for hydraulic-support face-guard pose estimation and shearer cutting-interference warning in fully mechanized coal mining faces. The method integrates RGB, infrared, and LiDAR data; performs modality-specific preprocessing; establishes spatiotemporal registration; extracts Swin-T and BEV geometric features; adaptively fuses heterogeneous cues; estimates face-guard pose from 2D and 3D constraints; and converts the reconstructed geometry into a four-level warning state. On the additional 400-frame warning-evaluation set, the tri-modal system reached 96.5% overall warning accuracy. The clean calibration experiment had a 0.28 cm MAE and a 0.50 cm maximum absolute error, whereas the environmental-condition test had a maximum error of 2.20 cm under combined dust and mist and the raw pre-filter threshold-neighborhood diagnostic reached 2.80 cm. These results demonstrate the laboratory feasibility of multi-source visual fusion for active safety monitoring; equipment-level stopping distance, controller communication delay, actuator response, calibration drift, and field performance must be validated before operational deployment.

## Figures and Tables

**Figure 1:**
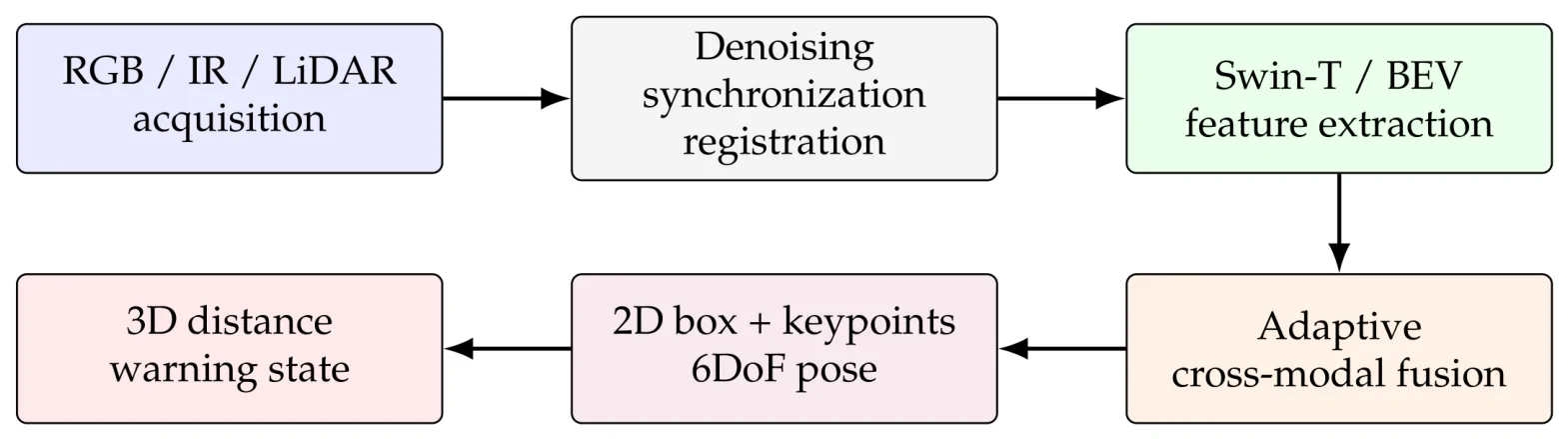
Overview of the proposed multi-source visual fusion pipeline for face-guard pose estimation and cutting-interference warning.

**Figure 2:**
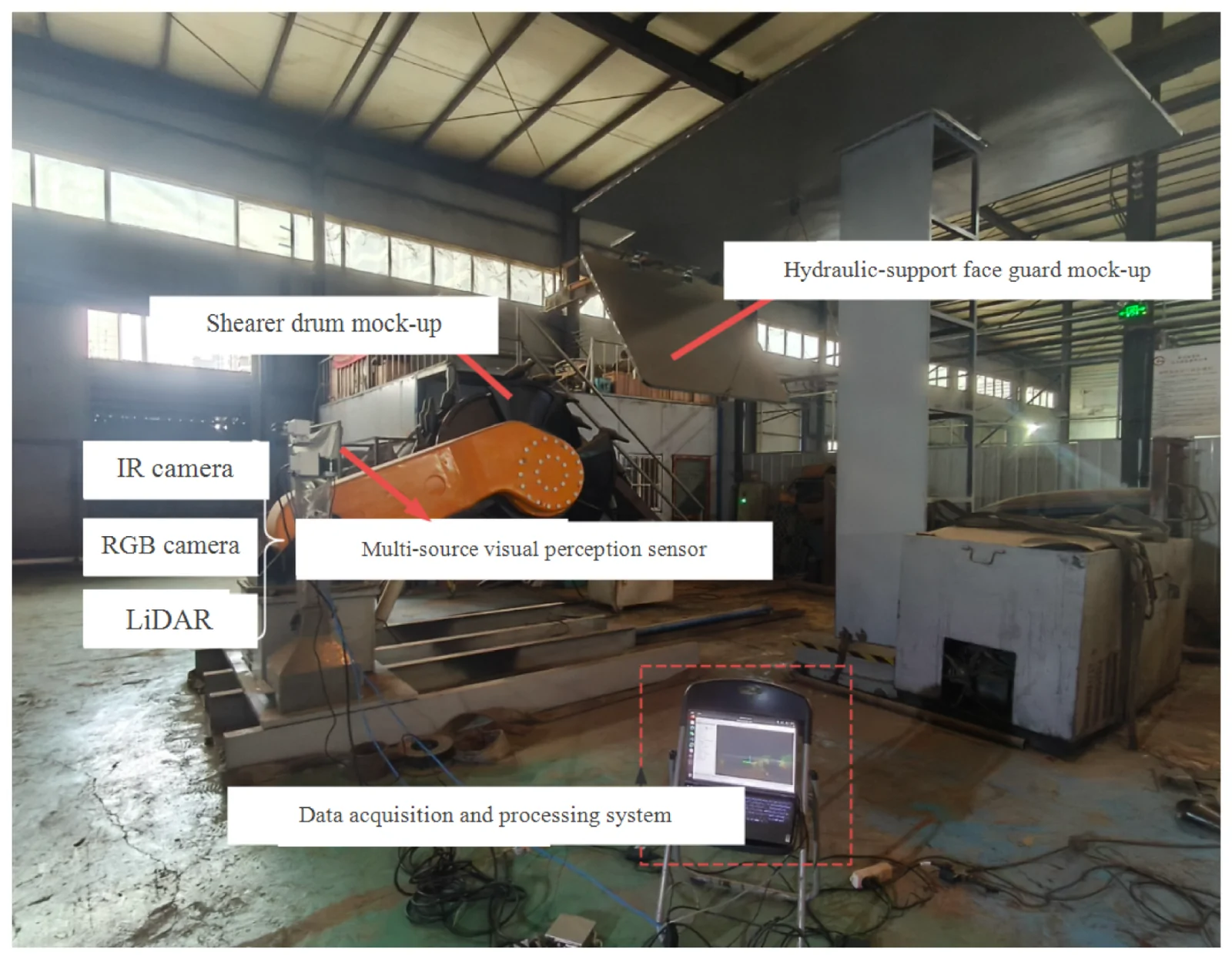
Experimental platform used for multimodal data acquisition and interference validation in the simulated fully mechanized mining-face environment.

**Figure 3:**
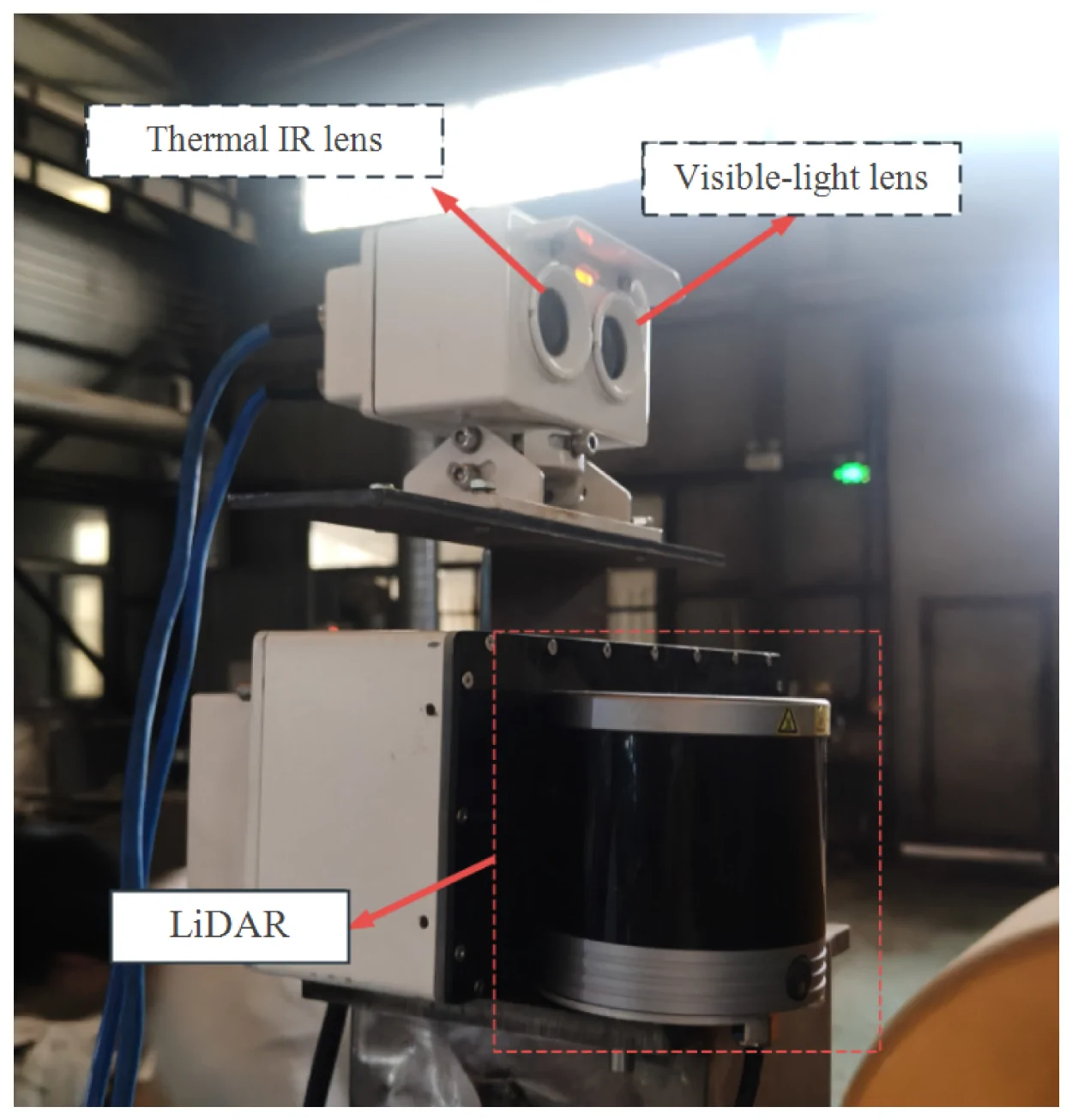
Co-mounted RGB/infrared camera and LiDAR setup used for synchronized acquisition.

**Figure 4:**
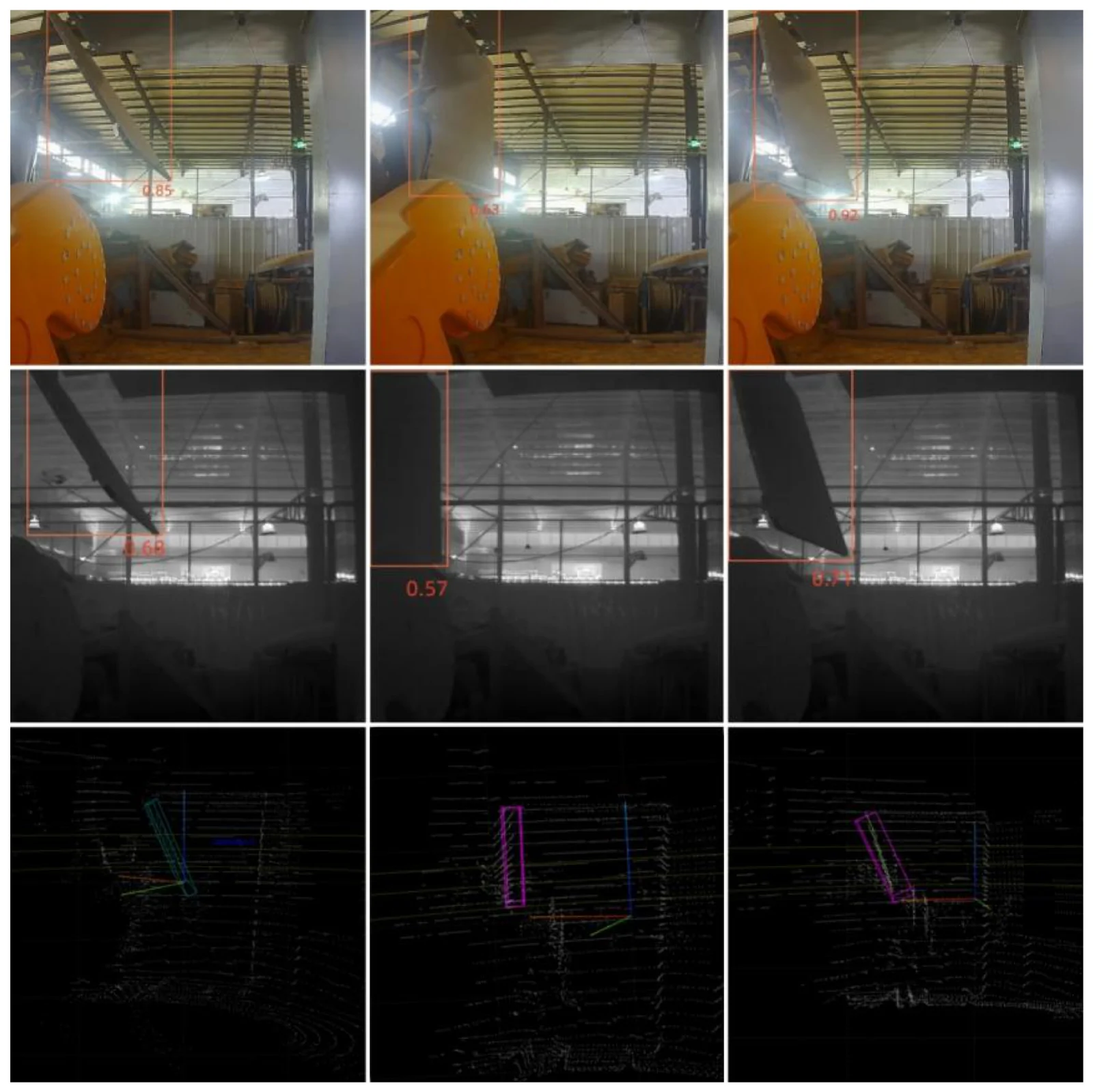
Representative multimodal detection comparison. The fused result preserves the face-guard outline and 3D geometry under a challenging mining-face scene.

**Figure 5:**
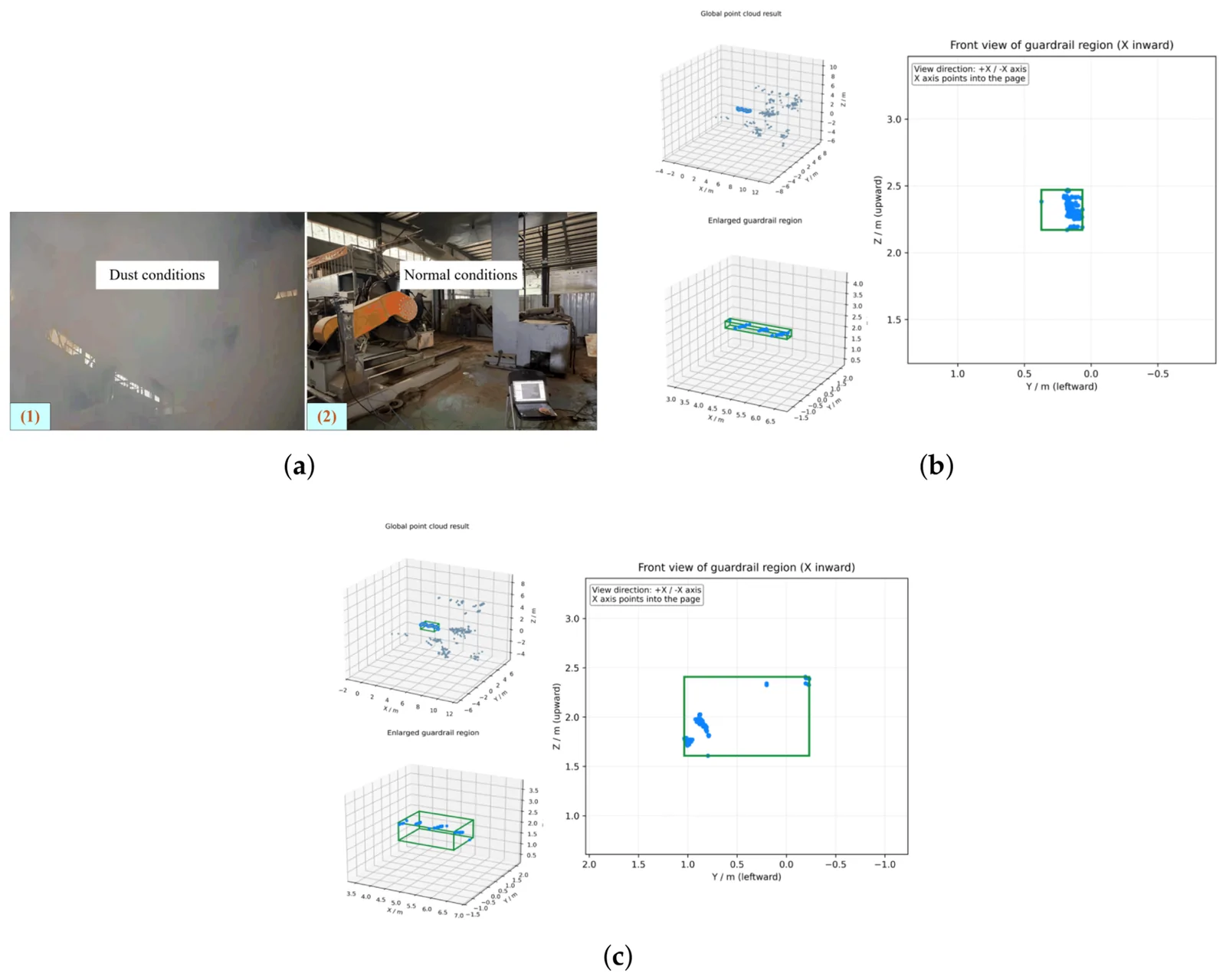
Qualitative stress cases for degradation and pose variation. (**a**) Dust and normal RGB scenes. (**b**) Sparse LiDAR returns at one guard pose. (**c**) Sparse returns with a changed guard orientation.

**Figure 6:**
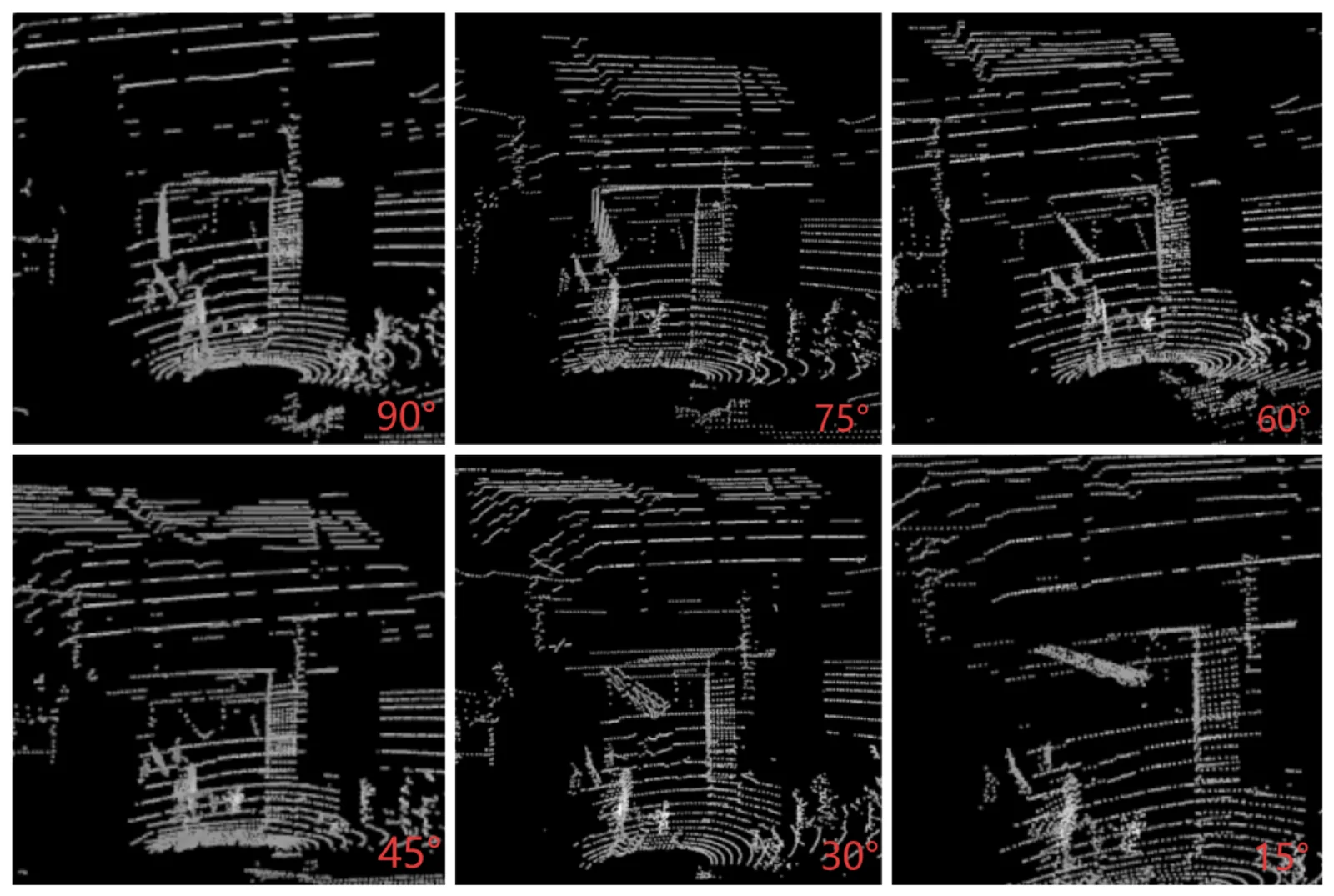
Representative pose sequence used for geometry recovery and distance evaluation.

**Table 1:** Sensor specifications and roles in the proposed system.

Sensor	Key Specification	Role
RGB camera	8-bit RGB, 1920 × 1080 output, 400 TVL, 0.05 lux low-light response	Texture, edges, and keypoints
Infrared camera	VOx uncooled FPA, 640 × 512, NETD < 50 mK, 25 fps	Thermal saliency under low light and dust
LiDAR	RoboSense RS-LiDAR-32, 360° horizontal FOV, approximately −25° to +15° vertical FOV, 0.33° minimum vertical resolution, 5/10/20 Hz	3D geometry and distance estimation

**Table 2:** Functional comparison of the sensing modalities in the mining-face environment.

Property	RGB Camera	Infrared Camera	LiDAR
Illumination dependence	High; image contrast changes with local lighting	Low; passive thermal response	Low; active ranging
Dust/mist sensitivity	Strong scattering and texture loss	More stable foreground saliency	Outliers and attenuation may occur
Texture/semantic cue	Rich color and edge information	Thermal gradient and foreground cue	Limited semantic cue
3D geometry	Indirect, projection-dependent	Indirect, low spatial detail	Direct metric geometry
Primary role in this study	2D boundary and keypoint evidence	Degraded-environment compensation	Pose and clearance support

**Table 3:** Dataset composition and annotation summary.

Item	Setting
Total synchronized frames	2400
Standard groups	6
Frames per group	400
Frame-level train/validation/test split	1680/480/240
Modalities	RGB, infrared, LiDAR
Annotations	2D bounding boxes, corner keypoints, 3D pose targets
Annotation tool	SUSTechPOINTS [12]

**Table 4:** Experimental condition design for the multimodal dataset.

Factor	Setting and Purpose
Face-guard pose	Multiple extension angles were used to cover the transition from retracted to near fully extended states and to generate different clearance ranges.
Ranging-arm pitch	Low, middle, and high pitch states were included to change the relative position of the shearer dangerous region.
Environmental condition	Normal, dust, mist, and dust–mist cases were arranged to test the degradation and complementarity of RGB, infrared, and LiDAR observations.
Frame organization	Six standard frame groups were retained after screening, with 400 synchronized multimodal frames per group.
Evaluation focus	Detection, keypoint localization, pose recovery, distance error, warning classification, and runtime were evaluated on the same platform.

**Table 5:** Warning levels and system responses.

Level	Distance Range	System Response
L0	$D_{t}>300$ mm	Safe operation, no intervention
L1	150–300 mm	Critical warning, attention required
L2	50–150 mm	Trend warning, speed reduction and adjustment
L3	$D_{t}\leq 50$ mm	Interference warning, emergency interlock

**Table 6:** Detection performance of different modality combinations.

Configuration	Metric	Result
RGB only	mAP@0.5	89.2%
Infrared only	mAP@0.5	83.6%
RGB + infrared	mAP@0.5	92.4%
RGB + infrared + LiDAR	mAP@0.5	98.5%
Tri-modal system	Runtime	Approximately 10 Hz
Tri-modal system	3D keypoint localization MAE	1.40 cm

**Table 7:** Representative baseline comparison on the held-out frame-level test set.

Method	mAP@0.5	mAP@0.5:0.95	Trans. MAE (cm)	Rot. MAE (°)	Params (M)	GFLOPs	Latency (ms)
Geometry-only envelope	61.8%	38.6%	4.8	4.6	0	3.1	12
RGB-only FCOS	89.2%	72.4%	3.9	3.4	28.1	8.7	52
LiDAR-only PointNet++	87.6%	66.3%	2.9	2.6	1.5	4.9	38
Early concatenation	94.1%	75.8%	2.0	1.9	53.4	16.2	118
Late decision fusion	95.4%	77.2%	1.8	1.7	55.1	17.4	126
Camera–LiDAR deep fusion	96.0%	78.4%	1.7	1.6	48.2	14.9	111
Proposed adaptive axial fusion	98.5%	84.7%	1.2	1.1	44.6	13.5	96

**Table 8:** Fusion-module ablation on the held-out frame-level test set.

Configuration	mAP@0.5	Trans. MAE (cm)	Rot. MAE (°)	Latency (ms)
Full adaptive axial fusion	98.5%	1.2	1.1	96
Equal-weight fusion	96.9%	1.7	1.7	93
Without axial attention	97.2%	1.5	1.4	88
Without temporal filtering	96.4%	1.8	1.9	94
Without preprocessing	94.8%	2.3	2.4	79

**Table 9:** Main technical challenge analysis and intended mitigation.

Case	Observable Symptom	Solutions
Dust or mist	RGB contrast and LiDAR return completeness decrease	Infrared quality cue, statistical filtering, and adaptive modality weighting
Sparse LiDAR	Guard plane contains gaps or isolated returns	Image keypoints initialize pose; valid LiDAR points constrain metric depth
Partial occlusion	One or more corners become unreliable	Robust reprojection loss and previous-pose temporal prior
Registration error	Projected image boundary and point cloud disagree	Calibration residual monitoring and NDT–ICP refinement
Near threshold	Small distance fluctuation changes the raw level	±20 mm hysteresis and three-frame confirmation

**Table 10:** 6DoF pose validation on the held-out frame-level test set.

Quantity	MAE	RMSE	Maximum
Translation error	1.20 cm	1.62 cm	3.90 cm
Rotation error	1.10°	1.48°	3.80°
Corner reprojection error	2.7 px	3.4 px	7.1 px
3D keypoint localization error	1.40 cm	1.76 cm	3.60 cm

**Table 11:** Representative distance measurement results in the calibration experiment.

No.	Ref. (cm)	Est. (cm)	Abs. (cm)	No.	Ref. (cm)	Est. (cm)	Abs. (cm)
1	4.0	4.2	0.2	11	33.1	33.4	0.3
2	7.0	7.1	0.1	12	36.7	36.2	0.5
3	9.5	9.4	0.1	13	39.2	38.9	0.3
4	11.3	11.4	0.1	14	43.5	43.8	0.3
5	14.2	14.4	0.2	15	46.8	46.4	0.4
6	17.6	17.5	0.1	16	51.2	51.7	0.5
7	21.5	21.3	0.2	17	54.7	54.3	0.4
8	24.8	24.9	0.1	18	58.4	58.0	0.4
9	27.3	27.1	0.2	19	61.9	62.4	0.5
10	30.6	30.4	0.2	20	64.3	63.8	0.5

**Table 12:** Distance estimation error under representative environmental conditions; the maximum values refer to each listed environmental condition.

Condition	MAE (cm)	RMSE (cm)	SD (cm)	Maximum (cm)
Normal environment	0.42	0.55	0.36	1.20
Light dust	0.56	0.71	0.44	1.50
Light mist	0.63	0.82	0.51	1.60
Dust + mist	0.91	1.16	0.73	2.20

**Table 13:** Raw pre-filter distance errors and warning-level changes near the three decision thresholds.

Threshold	Evaluation Band	MAE (cm)	RMSE (cm)	Maximum (cm)	Raw Label Changes
50 mm	30–70 mm	0.72	0.91	2.00	1/40
150 mm	130–170 mm	0.84	1.05	2.30	2/40
300 mm	280–320 mm	1.03	1.28	2.80	2/40

**Table 14:** Confusion matrix for the four-level warning policy on the additional 400-frame warning-evaluation set.

Reference/Predicted	L0	L1	L2	L3
L0	195	5	0	0
L1	2	95	3	0
L2	0	2	57	1
L3	0	0	1	39

**Table 15:** Precision, recall, and F1-score for the four warning levels.

Class or Aggregate	Precision (%)	Recall (%)	F1-Score (%)	Support
L0	98.98	97.50	98.24	200
L1	93.14	95.00	94.06	100
L2	93.44	95.00	94.21	60
L3	97.50	97.50	97.50	40
Macro average	95.77	96.25	96.00	400
Overall accuracy	–	–	96.50	400
L2/L3 safety recall (not L0)	–	100.00	–	100
L2/L3 exact-level recall	–	96.00	–	100

## Data Availability

The synchronized multimodal data used in this study are available on request from the corresponding author.
